# Efficacy of intra-articular ketorolac for pain control in arthroscopic surgeries: a systematic review and meta-analysis

**DOI:** 10.1186/s13018-021-02833-4

**Published:** 2021-11-22

**Authors:** Jingjing Yang, Bin Ni, Xiaoyan Fu

**Affiliations:** 1 Department of Pharmacy, Hangzhou Third People’s Hospital, 38 Xihu Ave, Shangcheng District, Hangzhou, 310009 Zhejiang Province China; 2 Department of Pharmacy, Affiliated Hangzhou First People’s Hospital, Zhejiang University School of Medicine, 261 Huansha Road, Shangcheng District, Hangzhou, 310003 Zhejiang Province China

**Keywords:** Arthroscopy, Knee joint, Shoulder joint, Pain, NSAIDs

## Abstract

**Background:**

The current systematic review and meta-analysis aimed to synthesize evidence on the efficacy of intra-articular ketorolac for patients undergoing arthroscopic surgeries.

**Methods:**

PubMed, Embase, ScienceDirect, and Google Scholar databases were searched for randomized controlled trials assessing the analgesic effect of intra-articular ketorolac for arthroscopic surgery of hip/knee or shoulder joint.

**Results:**

Six studies were included. Two studies were on shoulder arthroscopy, while others were on knee joint. Meta-analysis revealed that patients receiving intra-articular ketorolac had significantly lower pain scores at 2–4 h (MD: − 0.58 95% CI: − 0.88, − 0.19 *I*^2^ = 49% *p* = 0.002), 6–8 h (MD: − 0.77 95% CI: − 1.11, − 0.44 *I*^2^ = 31% *p* < 0.00001), 12 h (MD: − 0.94 95% CI: − 1.21, − 0.67 *I*^2^ = 0% *p* < 0.00001), and 24 h (MD: − 1.28 95% CI: − 1.85, − 0.71 *I*^2^ = 84% *p* < 0.00001) as compared to the control group (Certainty of evidence: low-moderate). Analysis of three studies revealed a tendency of reduced analgesic consumption in patients receiving intra-articular ketorolac, but the difference did not reach statistical significance (MD: − 0.53 95% CI: − 1.07, 0.02 *I*^2^ = 55% *p* = 0.06).

**Conclusions:**

Preliminary evidence from a limited number of studies indicates that additional intra-articular ketorolac to multimodal analgesia results in reduced pain scores up to 24 h after arthroscopic surgery. The clinical relevance of small changes in pain scores is debatable. Also, scarce data suggest that consumption of analgesics may not be reduced with intra-articular ketorolac. Since pain scores can be influenced by the primary diagnosis and dose of ketorolac, the results should be interpreted with caution. The certainty of the evidence is low-moderate. There is a need for future RCTs to further strengthen current evidence.

**Supplementary Information:**

The online version contains supplementary material available at 10.1186/s13018-021-02833-4.

## Introduction

With the invention of minimally invasive techniques, arthroscopic surgery has become the standard of care for managing joint disorders. Arthroscopy can either be used to visualize the joint for diagnostic purposes, manipulation of tissues, or introduction of biologic therapies [1]. Indeed, the spectrum of indications for arthroscopic surgery has expanded rapidly in the past decade and it now includes a variety of conditions like synovitis, ligament tears, loose bodies, and tissue impingement [2]. The technique is commonly used for the knee, hip, and shoulder joints and is being increasingly used for smaller joints like the ankle, elbow, and wrist [3–5]. Arthroscopy has revolutionized the management of intra-articular soft tissue procedures without the need for open arthrotomies, thereby reducing postoperative morbidity and improving rehabilitation [6]. Despite being a minimally invasive procedure, postoperative pain can be a significant problem after arthroscopic surgery and inadequate analgesia can increase time to rehabilitation, lengthen hospital stay and subsequently increase healthcare costs [7, 8].

A variety of peripheral nerve blocks, peri- or intra-articular analgesics/anesthetics, and oral/parenteral analgesics have been used for pain control with arthroscopic procedures [9]. However, despite intense research, the most optimal regional anesthetic technique for pain control is still debatable [10]. Amongst the different modalities, intra-articular injections have the advantage of acting directly at the site of surgery thereby producing a potent analgesic response. Intra-articular injections of opioid analgesics like morphine have been commonly used for pain control after arthroscopy [11]. However, evidence of its efficacy has been questionable [12]. In search of other analgesic options, researchers have also used non-steroidal anti-inflammatory drugs (NSAIDs) for pain control after arthroscopic surgery. Amongst NSAIDs, ketorolac, a carboxylic acid derivative has been successfully used for control of mild to severe pain with an analgesic efficacy similar to opioids but with reduced adverse events [13]. In the past, several studies have assessed the efficacy of intra-articular ketorolac for pain control after arthroscopic surgeries [14–16]. However, to the best of our knowledge, no study has systematically reviewed the evidence on its effectiveness. Recently, Wan et al. [17] have reviewed the impact of ketorolac supplementation for analgesia after knee arthroscopy, but their study combined data of intra-articular as well as oral or parenteral ketorolac. Given such deficiency in the literature, we aimed to conduct a systematic literature search and pool evidence on the efficacy of intra-articular ketorolac for patients undergoing arthroscopic surgeries. The research question to be answered was: “Does intra-articular ketorolac given singularly or as a part of multimodal analgesic protocol reduce pain and analgesic consumption in patients undergoing arthroscopic surgeries?”.

## Material and methods

This systematic review and meta-analysis was carried out according to the guidelines of the PRISMA statement (preferred reporting items for systematic reviews and meta-analyses) [18] and the Cochrane Handbook for Systematic Reviews of Intervention [19]. The PROSPERO registration number of the study is CRD42021268402.

### Literature search

Two reviewers independently searched the electronic databases of PubMed, Embase, ScienceDirect, and Google Scholar for relevant articles. The search strategy was formalized with the aid of a medical librarian, and the search limits were set from inception to August 1, 2021. No language restriction was applied. The keywords used for the literature search included: “ketorolac”, “intra-articular”, “arthroscopy”, and “arthroscopic surgery”. Details of the literature search common to all databases are presented in Additional file 1: Table S1. The primary search results were assessed initially by their titles and abstracts to identify citations requiring full-text analysis. The full texts of the articles were reviewed by the two reviewers independently based on the inclusion and exclusion criteria. Any disagreements were resolved by discussion. We also carried out manual scoping of the bibliography in included studies for any additional articles.

### Inclusion criteria

Eligibility criteria for this review were structured using the PICOS (population, intervention, comparison, outcome, and study design) framework. Details are as follows:

#### Population

Patients undergoing any type of arthroscopic surgery of a major joint (knee, hip, or shoulder).

#### Intervention

Use of intra-articular ketorolac after completion of arthroscopic surgery. Ketorolac could have been the sole analgesic or adjuvant to other drugs.

#### Comparison

No injection or injection with placebo (normal saline) along with other baseline analgesic used in the intervention group.

#### Outcomes

Pain scores, postoperative analgesic consumption, or adverse events.

#### Study design

Randomized controlled trials (RCTs).

Exclusion criteria were: (1) studies administering oral or intravenous ketorolac; (2) studies comparing ketorolac with other analgesic drugs; (3) studies not reporting outcome data as either mean ± standard deviation (SD), median {interquartile range] or graphically; (4) non-RCTs, abstracts, editorials, review articles, and case reports.

### Data extraction and risk of bias assessment

A data extraction sheet was used by two reviewers to extract relevant data from the studies. Details of the first author, publication year, study location, joint studied, sample size, age and gender details, duration of surgery, injection protocol of ketorolac and control group, use of other analgesic drugs, and study outcomes were extracted. The outcomes of interest for our review were pain scores at rest on the Visual Analog Scale (VAS), analgesic consumption in the postoperative period, and adverse events.

Two reviewers independently assessed the quality of included RCTs using the Cochrane Collaboration’s risk of bias assessment tool-2 [19]. Every study was assessed for randomization process, deviation from intended intervention, missing outcome data, measurement of outcomes, and selection of reported results. Based on the risk of bias in individual domains, the overall bias was marked as “high risk”, “some concerns”, or “low risk”. Any disagreements related to data extraction or quality assessment were resolved by discussion. The certainty of the evidence was assessed using the Grading of Recommendations Assessment, Development, and Evaluation (GRADE) tool using the GRADEpro GDT software [GRADEpro Guideline Development Tool. McMaster University, 2020 (developed by Evidence Prime, Inc.)].

### Statistical analysis

The software “Review Manager” (RevMan, version 5.3; Nordic Cochrane Centre [Cochrane Collaboration], Copenhagen, Denmark; 2014) was used for the meta-analysis. We summarized pain scores at different time intervals with the mean difference (MD) and 95% confidence intervals (CI). Since there was dissimilarity between studies on the type of rescue analgesic, data for the same were summarized using standardized mean difference (SMD). When data were only presented graphically, Engauge Digitizer version 12.1 was used to extract numerical data. Median, range, and interquartile range data were converted into mean and standard deviation (SD) when required using the method of Wan et al. [20]. Data on adverse events were to be pooled using risk ratios (RR). The random-effects model was used for all the meta-analyses. Heterogeneity was assessed using the *I*^2^ statistic. *I*^2^ values of 25–50% represented low, values of 50–75% medium, and more than 75% represented substantial heterogeneity. Due to a limited number of studies in the meta-analysis (less than 10), funnel plots were not used to assess publication bias. We conducted a sensitivity analysis for the meta-analysis of pain scores. In the analysis, individual studies were excluded one at a time and the effect size was recalculated for the remaining studies in the meta-analysis software itself.

## Results

### Details of included studies

**Fig. 1:**
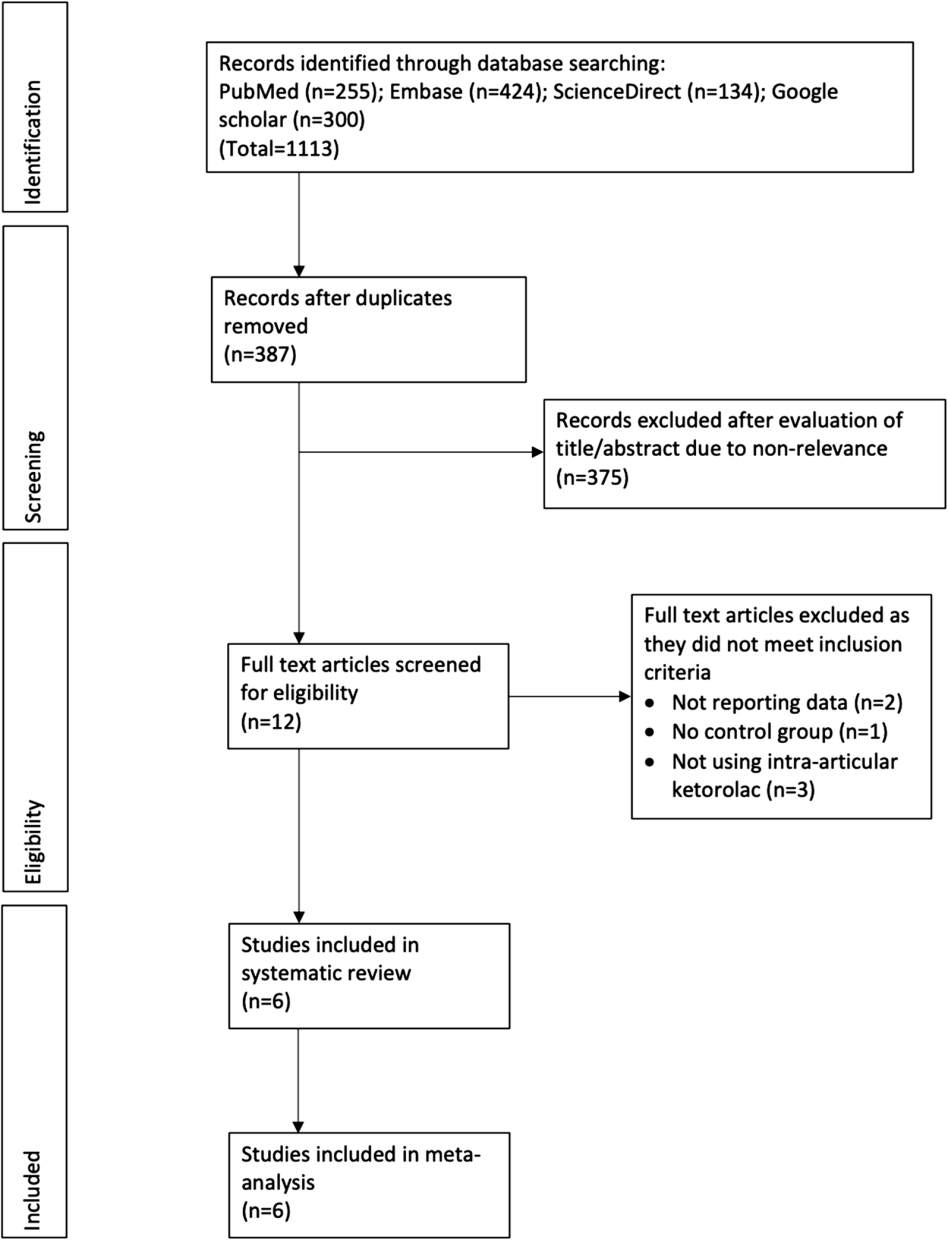
Study flowchart

The number of search results at each stage is summarized in Fig. 1. A total of six RCTs fulfilled the inclusion criteria and were analyzed in this review [14–16, 21–23]. Details of included studies are presented in Table 1. Three studies [15, 16, 23] were conducted in Asia and three in Europe [14, 21, 22]. Except for two [15, 23], all studies were on the knee joint. The majority of the studies used intra-articular ketorolac either as an adjunct to other intra-articular or oral analgesics. There was a wide variation in the dosage of ketorolac used.

**Table 1:** Details of included studies

Study	Location	Joint	Intra-articular ketorolac protocol	Control protocol	Sample size		Mean age		Male gender (%)		Operation time (mins)		Other analgesic drugs
					K	C	K	C					
Xu et al. [15]	China	Shoulder	20 ml of solution containing ketorolac 10 mg, ropivacaine 450 mg, morphine 5 mg, triamcinolone 25 mg	20 ml of solution containing ropivacaine 450 mg, morphine 5 mg, triamcinolone 25 mg	30	30	51.8 ± 6.9	53.5 ± 6.3	33.3	43.3	53.9 ± 12.5	55.8 ± 14.7	Imrecoxib 100 mg oral twice daily till discharge
Solheim et al. [14]	Norway	Knee	5 ml of normal saline containing ketorolac 5 mg	5 ml of normal saline	22	20	43 ± 12	50 ± 12	45.5	45	NR	NR	None
Rohktabnak et al. [16]	Iran	Knee	0.5% ropivacaine (150 mg) and ketorolac 30 mg in 30 ml saline	0.5% ropivacaine (150 mg) in 30 ml saline	20	20	42.4 ± 12.2	45.1 ± 13.6	85	70	38.7 ± 9.7	39.5 ± 9.6	Oral PCM 1 g 8 hourly if VAS > 3, if insufficient relief tramadol 50 mg maximum four times a day
Kim et al. [23]	South Korea	Shoulder	0.5% ropivacaine 100 ml, fentanyl 10 µg/kg, Ketorolac 150 mg through catheter at 2 ml/hr with bolus dose of 0.5 ml for 48 h	0.5% ropivacaine 100 ml, fentanyl 10 µg/kg, through catheter at 2 ml/hr with bolus dose of 0.5 ml for 48 h	10	10	41.5 ± 12.3	47.7 ± 8.9	70	70	NR	NR	NR
Vintar et al. [22]	Sweden	Knee	0.25% ropivacaine, morphine 0.2 mg/mL and ketorolac 1 mg/mL in 100 ml solution via catheter with 10 ml bolus	0.25% ropivacaine, morphine 0.2 mg/mL in 100 ml solution via catheter with 10 ml bolus	13	13	27 ± 9	35 ± 6	55	50	88 ± 6	89 ± 16	Oral PCM 1 g 6 hourly and IV morphine PCA
Calmet et al. [21]	Spain	Knee	Ketorolac 60 mg	10 ml saline	20	20	NR	NR	NR	NR	NR	NR	Oral PCM 650 mg 6 hourly and tramadol 50 mg if VAS > 5

PCM, paracetamol; VAS, visual analog scale; PCA, patient-controlled analgesia; NR, not reported; K, ketorolac group; C, control group

The risk of bias analysis of the included studies as per the authors’ judgment is presented in Table 2. Four studies were of high quality with a low overall risk of bias [14–16, 22]. The overall risk of bias was marked as “some concerns” for the remaining two studies [21, 23].

**Table 2:** Risk of bias in included studies

Study	Randomization process	Deviation from intended intervention	Missing outcome data	Measurement of outcomes	Selection of reported result	Overall risk of bias
Xu et al. [15]	Low risk	Low risk	Low risk	Low risk	Low risk	Low risk
Solheim et al. [14]	Low risk	Low risk	Low risk	Low risk	Low risk	Low risk
Rohktabnak et al. [16]	Low risk	Low risk	Low risk	Low risk	Low risk	Low risk
Kim et al. [23]	Some concerns	Low risk	Low risk	Some concerns	Low risk	Some concerns
Vintar et al. [22]	Low risk	Low risk	Low risk	Low risk	Low risk	Low risk
Calmet et al. [21]	Some concerns	Low risk	Low risk	Low risk	Low risk	Some concerns

### Meta-analysis

**Fig. 2:**
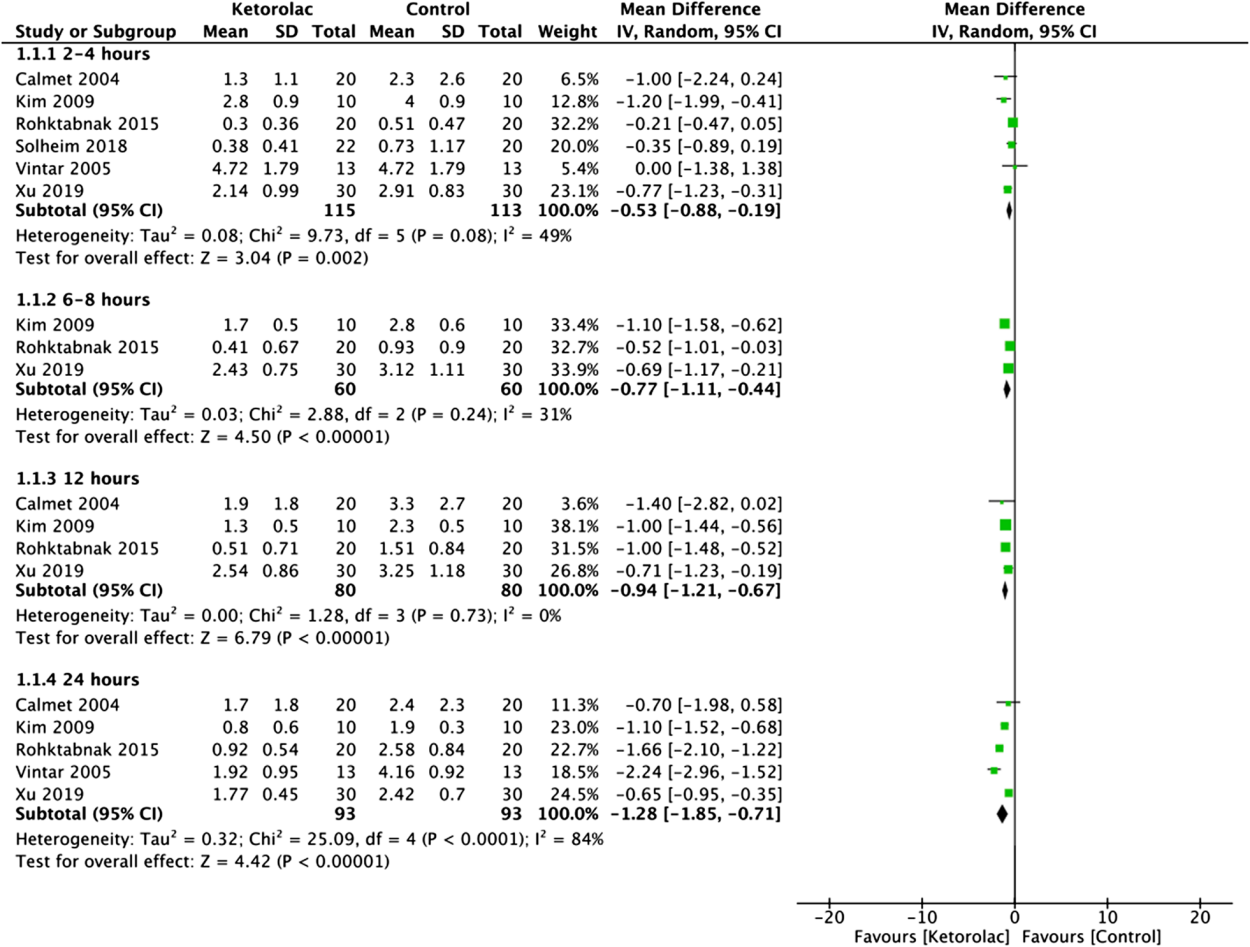
Meta-analysis of pain scores between ketorolac and control groups at different time intervals

Data on pain scores were grouped according to different time intervals as 2–4 h, 6–8 h, 12 h, and 24 h after surgery. Meta-analysis revealed that patients receiving intra-articular ketorolac had significantly lower pain scores at 2–4 h (MD: − 0.58 95% CI: − 0.88, − 0.19 *I*^2^ = 49% *p* = 0.002), 6–8 h (MD: − 0.77 95% CI: − 1.11, − 0.44 *I*^2^ = 31% *p* < 0.00001), 12 h (MD: − 0.94 95% CI: − 1.21, − 0.67 *I*^2^ = 0% *p* < 0.00001), and 24 h (MD: − 1.28 95% CI: − 1.85, − 0.71 *I*^2^ = 84% *p* < 0.00001) as compared to the control group (Fig. 2). On sensitivity analysis, there was no change in the significance of the results on the exclusion of any study. The certainty of the evidence was low-moderate according to GRADE (Additional file 2: Table S2).

**Fig. 3:**
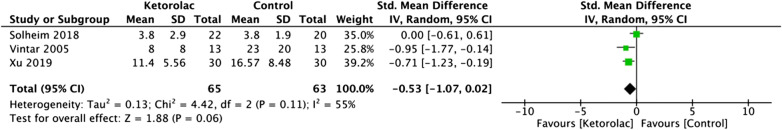
Meta-analysis of consumption of rescue analgesic between ketorolac and control groups

Data on the consumption of rescue analgesia were reported just by three studies. On pooled analysis, we noted a tendency of reduced rescue analgesic consumption in patients receiving intra-articular ketorolac, but the difference did not reach statistical significance (MD: − 0.53 95% CI: − 1.07, 0.02 *I*^2^ = 55% *p* = 0.06) (Fig. 3). The certainty of the evidence was moderate according to GRADE (Additional file 2: Table S2).

Incidence of adverse events was not universally reported by the included studies. Amongst the studies reporting the same, Xu et al. [15] found no statistically significant difference in the risk of between ketorolac and control groups. Vintar et al. [22] also noted no difference in the risk of PONV, pruritus, urinary retention, or dizziness/vertigo between the two groups. In the absence of data, a meta-analysis was not carried out for adverse events.

## Discussion

This study is the first systematic review and meta-analysis evaluating the efficacy of intra-articular ketorolac for pain control after arthroscopic surgeries. Pooled data from a limited number of studies indicate that pain scores up to 24 h are significantly reduced with the addition of intra-articular ketorolac to multimodal analgesic protocol. Data from a few studies also indicated that analgesic consumption is reduced in the postoperative period with intra-articular ketorolac.

Traditionally, opioids have been the mainstay of pain control after most surgical procedures. However, owing to several associated side-effects like nausea, vomiting, dizziness, and urinary retention along with the risk of future opioid dependence, there has been a consistent effort by the surgical community to reduce opioid usage [24]. Nevertheless, this has to be carefully balanced against adequate analgesia as uncontrolled pain can lead to increased length of hospital stay and reduced patient satisfaction [7, 8]. The enhanced recovery protocol after surgery also indicates that the use of preemptive multimodal analgesia reduces pain, opioid use, and adverse events in arthroscopic surgery [25]. In this context, intra-articular injections are an attractive option for clinicians as it anticipates and prevents the onset of pain thereby reducing the need of systemic analgesia [16].

The use of ketorolac as an intra-articular injection stems from the fact that it is one of the few NSAIDs available as a parenteral preparation. Due to its proven efficacy as a potent analgesic, the drug has been also used as an intravenous (IV) agent for pain control after arthroscopic surgeries [26, 27]. However, it is postulated that intra-articular administration might result in a greater analgesic effect and reduce the incidence of systemic adverse events like gastrointestinal symptoms, platelet inhibition, and renal dysfunction [15]. In our meta-analysis, we noted a statistically significant reduction of pain scores with the use of intra-articular ketorolac at all time points ranging from 2–4 h to 24 h. The results were consistent on sensitivity analysis as there was no change in the direction of significance with the exclusion of any study. While the analgesic effect of ketorolac is mainly derived from its inhibition of prostaglandins, the prolonged action up to 24 h can be attributed to its high protein binding which causes delayed action of the drug [28]. Furthermore, since ketorolac is free from respiratory and central nervous system effects, it has a distinct advantage over narcotic analgesics [13]. Calmet et al. [21] in their small study have demonstrated that intra-articular ketorolac provides better analgesia as compared to intra-articular bupivacaine and morphine.

Nevertheless, the pain outcomes should be interpreted with caution given the values of the MD in our analysis. In 1989, Jaeschke et al. [29] conceptualized the principle of “minimally important clinical difference” (MCID) which was defined as “the smallest difference in score in the domain of interest which participants perceive as beneficial and which would mandate, in the absence of troublesome side effects and costs, a change in the patient’s management”. This concept stresses the fact that the difference in pain scores achieved by the drug should be clinically relevant to the patient even if the MD is statistically significant [30]. In our meta-analysis, the MD in pain scores ranged from 0.58 at 2–4 h to 1.28 at 24 h. Indeed, the difference is not large and the clinical relevance of reduced pain with ketorolac can be questionable. In a recent study, Laigaard et al. [31] have shown that MCID for pain after hip/knee arthroplasty is 1.5 on VAS. Olsen et al. [30] in their systematic review have suggested that MCID can range from 0.8 to 4 for acute pain and can vary with baseline pain, definitions of improved patients, and study design. Due to the scarcity of data on MCID for early pain after arthroscopic surgeries, the clinical relevance of analgesia offered by intra-articular ketorolac cannot be judged.

In the second part of our meta-analysis, we noted no statistically significant difference in analgesic consumption in patients receiving intra-articular ketorolac. While there was a tendency for reduced analgesic consumption in the ketorolac group, a significant limitation of this analysis was that just three studies reported data on analgesic consumption. Indeed, analgesic consumption can be considered as a surrogate marker of pain control and reduced postoperative opioid consumption is beneficial as it leads to reduced opioid-related adverse events like respiratory depression, PONV, pruritus, and urinary retention. However, due to the scarcity of data, we could not analyze the incidence of drug-related adverse events in our review. The results of our study concur with the previous review of Wan et al. [17] which reported significantly reduced pain scores and time to the first analgesic request in patients receiving ketorolac supplementation but no difference in analgesic consumption or incidence of nausea and vomiting. However, important limitations of their review were the combined analysis of data of IV and intra-articular ketorolac and the inclusion of one retracted article [32].

The limitations of our review need to be specified. Foremost, the availability of just six studies is a significant hindrance for strong conclusions. The majority of included studies were of small sample size, resulting in the reduced statistical power of our analysis. Furthermore, the study outcomes were not universally reported by all studies and just three trials could be included in the analysis of total analgesic consumption. Second is the high methodological heterogeneity amongst the studies. There were differences in the joint studied, the type of surgery, the dosage of ketorolac, and the baseline analgesic protocol all of which could have skewed the study results.

The strength of our study is that for the first time in the literature we have pooled evidence on the efficacy of intra-articular ketorolac for pain control after arthroscopic surgery. Unlike the previous review [17], studies only on intra-articular administration of ketorolac were included to provide clarity on its efficacy. Since prior published studies on intra-articular ketorolac are of a small sample size, the current review comparing 115 patients with intra-articular ketorolac and 113 controls presents the best possible evidence to clinicians using this drug in routine practice.

## Conclusions

Preliminary evidence from a limited number of studies indicates that additional intra-articular ketorolac to multimodal analgesia results in reduced pain scores up to 24 h after arthroscopic surgery. The clinical relevance of small changes in pain scores is debatable. Also, scarce data suggest that consumption of analgesics may not be reduced with intra-articular ketorolac. Since pain scores can be influenced by the primary diagnosis and dose of ketorolac, the results should be interpreted with caution. The certainty of the evidence is low-moderate. There is a need for future RCTs comparing intra-articular ketorolac with placebo and other analgesic drugs to further strengthen current evidence.

## Supplementary Information

**Additional file 1: **Search strategy.

**Additional file 2:** GRADE assessment of evidence.

## Data Availability

The datasets used and/or analyzed during the current study are available from the corresponding author on reasonable request.

## Abbreviations

PRISMA: Preferred reporting items for systematic reviews and meta-analyses
PICOS: Population, intervention, comparison, outcome, and study design
RCTs: Randomized controlled trials
SD: Standard deviation
CI: Confidence intervals
SMD: Standardized mean difference
RR: Risk ratios
PONV: Postoperative nausea and vomiting
IV: Intravenous
