# Supplementary material for: Efficacy of intra-articular ketorolac for pain control in arthroscopic surgeries: a systematic review and meta-analysis
Source: J Orthop Surg Res. 2021 Nov 22;16:688. doi: 10.1186/s13018-021-02833-4 (PMC8607634; doi:10.1186/s13018-021-02833-4)
Supplement: Supplementary file 1 — Additional file 1: Search strategy. [file 13018_2021_2833_MOESM1_ESM.docx]

Supplementary Table 1: Search strategy

| **Search number** | **Query** | **Search Details** |
| --- | --- | --- |
| **1** | (ketorolac) AND (arthroscopy) | ("ketorolac"[MeSH Terms] OR "ketorolac"[All Fields]) AND ("arthroscopy"[MeSH Terms] OR "arthroscopy"[All Fields] OR "arthroscopies"[All Fields]) |
| **2** | (ketorolac) AND (arthroscopic surgery) | ("ketorolac"[MeSH Terms] OR "ketorolac"[All Fields]) AND ("arthroscopy"[MeSH Terms] OR "arthroscopy"[All Fields] OR ("arthroscopic"[All Fields] AND "surgery"[All Fields]) OR "arthroscopic surgery"[All Fields]) |
| **3** | (intra-articular) AND (ketorolac) | "intra-articular"[All Fields] AND ("ketorolac"[MeSH Terms] OR "ketorolac"[All Fields]) |
