# Supplementary material for: Efficacy of intra-articular ketorolac for pain control in arthroscopic surgeries: a systematic review and meta-analysis
Source: J Orthop Surg Res. 2021 Nov 22;16:688. doi: 10.1186/s13018-021-02833-4 (PMC8607634; doi:10.1186/s13018-021-02833-4)
Supplement: Supplementary file 2 — Additional file 2: GRADE assessment of evidence. [file 13018_2021_2833_MOESM2_ESM.docx]

| **Supplementary table 2: GRADE assessment of evidence** | | | | | | | | | | | |
| --- | --- | --- | --- | --- | --- | --- | --- | --- | --- | --- | --- |
| **Certainty assessment** | | | | | | | **Summary of findings** | | | | |
| **Participants  (studies) Follow up** | **Risk of bias** | **Inconsistency** | **Indirectness** | **Imprecision** | **Publication bias** | **Overall certainty of evidence** | **Study event rates (%)** | | **Relative effect (95% CI)** | **Anticipated absolute effects** | |
|  |  |  |  |  |  |  | **With placebo** | **With Ketorolac** |  | **Risk with placebo** | **Risk difference with Ketorolac** |
| **Pain scores - 2-4 hours** | | | | | | | | | | | |
| 228 (6 RCTs) | serious ^a^ | not serious | not serious | not serious | none | ⨁⨁⨁◯ MODERATE | 113 | 115 | - | The mean pain scores - 2-4 hours was **0** | MD **0.58 lower** (0.88 lower to 0.19 lower) |
| **Pain scores - 6-8 hours** | | | | | | | | | | | |
| 120 (3 RCTs) | not serious | not serious | not serious | serious ^b^ | none | ⨁⨁⨁◯ MODERATE | 60 | 60 | - | The mean pain scores - 6-8 hours was **0** | MD **0.77 lower** (1.11 lower to 0.44 lower) |
| **Pain scores - 12 hours** | | | | | | | | | | | |
| 160 (4 RCTs) | serious ^a^ | not serious | not serious | serious ^b^ | none | ⨁⨁◯◯ LOW | 80 | 80 | - | The mean pain scores - 12 hours was **0** | MD **0.94 lower** (1.21 lower to 0.67 lower) |
| **Pain scores - 24 hours** | | | | | | | | | | | |
| 186 (5 RCTs) | serious ^a^ | not serious | not serious | not serious | none | ⨁⨁⨁◯ MODERATE | 93 | 93 | - | The mean pain scores - 24 hours was **0** | MD **1.28 lower** (1.85 lower to 0.71 lower) |
| **Rescue analgesic** | | | | | | | | | | | |
| 128 (3 RCTs) | not serious | not serious | not serious | serious ^b^ | none | ⨁⨁⨁◯ MODERATE | 63 | 65 | - | - | SMD **0.53 lower** (1.07 lower to 0.02 higher) |

**CI:** Confidence interval; **MD:** Mean difference; **SMD:** Standardised mean difference

#### Explanations

a. Some concerns for the studies of Kim et al an Calmet et al

b. limited number of studies with small sample size
